# Reduction Scrotoplasty With Concomitant Ventral Phalloplasty: Rethinking the Approach to a Common Urological Condition

**DOI:** 10.7759/cureus.79786

**Published:** 2025-02-27

**Authors:** Merary Z Nazario-Perez, Laura F Rodríguez-Fernández, Kenneth O Cintron-Cartagena, Javier Castillo

**Affiliations:** 1 Urology, Saint Luke’s Hospital, Ponce, PRI; 2 Surgery, Ponce Health Sciences University, Ponce, PRI; 3 Biochemistry, Ponce Health Sciences University, Ponce, PRI

**Keywords:** genital aesthetics, penile length perception, penoscrotal webbing, scrotal laxity, scrotal reduction surgery, scrotoplasty

## Abstract

Scrotal reduction surgery with concomitant ventral phalloplasty is a procedure designed to address increased scrotal laxity and penoscrotal webbing (PSW), conditions that can significantly impact genital aesthetics, patient comfort, and sexual satisfaction. In this case report, we present a 44-year-old man who developed persistent scrotal enlargement and PSW following bilateral hydrocelectomy. The patient experienced discomfort during daily activities and intercourse, along with a perception of decreased penile length. To achieve a more comprehensive correction, we utilized a novel modification of the transverse check mark-shaped incision, designed to address both scrotal laxity and PSW in a single-stage procedure. Unlike previous techniques that primarily focus on webbing excision, our approach incorporated additional modifications to optimize scrotal contouring and prevent residual laxity. This technique effectively resolved redundant scrotal tissue while optimizing both functional and cosmetic outcomes. The patient recovered without complications and reported significant improvement in scrotal contour and perceived penile length. As more cases of scrotal laxity are identified following urological procedures, surgeons and urologists should be aware of refined surgical techniques that can enhance patient satisfaction and quality of life.

## Introduction

The scrotum is a pouch-like structure divided into two compartments by the median raphe, extending from the anus through the perineum to the ventral side of the penis. Typically, the left side hangs lower than the right due to the longer spermatic cord. The scrotal walls are highly elastic and contain multiple layers, including rugated skin, superficial fascia (dartos), external spermatic fascia, the cremaster muscle, and internal spermatic fascia. The testicles are suspended within the scrotum, encased by the parietal and visceral layers of the tunica vaginalis, as well as the tunica albuginea [1].

While the scrotum’s elasticity supports the testes, changes in tissue integrity, such as excessive laxity or penoscrotal webbing (PSW), can cause functional and aesthetic concerns [1]. Scrotal laxity, defined as an enlarged scrotal sac hanging more than 1-2 cm below the tip of the penis, may develop due to aging or genetic factors or as a consequence of urological procedures [1]. Patients may experience discomfort in loose clothing, especially during physical activities such as walking and sports, or during intercourse. Additionally, increased scrotal laxity is often associated with persistent penoscrotal webbing.

Penoscrotal webbing (PSW) can be either congenital or, more commonly, an acquired condition. It is often a result of aggressive scrotal skin removal during circumcision. Currently, there is limited literature focused on PSW in adults, as most studies primarily address the pediatric population, where its prevalence is estimated at around 4% [2]. When PSW coexists with scrotal laxity, it can significantly impact sexual function and the psychological aspects of interpersonal relationships. This condition may cause difficulties in urinary function, sexual activity, and self-perception [1,3].

For individuals facing considerable distress or functional challenges due to scrotal laxity or PSW, surgical intervention is typically the main approach. Common reasons for considering surgery include discomfort during everyday activities, difficulties with intimacy, recurring infections, and significant psychological distress. While surgical correction is the dominant treatment method, some researchers have investigated the use of absorbable suspension sutures as a potentially less invasive alternative [4,5]. Numerous surgical techniques, such as tissue resection and reconstructive surgery, have been documented in the literature, all aimed at restoring both functionality and aesthetics, thereby improving patient satisfaction and overall quality of life [1,6].

In this context, we present a case involving a patient who developed significant scrotal laxity and PSW following bilateral hydrocelectomy. The patient reported significant discomfort and a subjective perception of reduced penile length. These concerns were managed through scrotal reduction surgery and ventral phalloplasty, using a modified novel transverse check mark-shaped incision. This case underscores the importance of recognizing post-surgical scrotal laxity and highlights the need for awareness of various surgical techniques that can improve patient outcomes.

## Case presentation

A 44-year-old man presented to the clinic with complaints of excessive scrotal skin, reporting discomfort and a reduced quality of life. He reported discomfort during physical activity and intercourse, as well as the perception of reduced penile length. His surgical history included a bilateral hydrocelectomy in September 2022, during which 1300 mL of straw-colored fluid was drained from his scrotal sac. At his 12-month follow-up, our patient exhibited scrotal laxity, which the literature defines as a scrotal sac hanging more than 1-2 cm below the tip of the penis [1]. In his case, the scrotum hung 5-6 cm below the tip of his flaccid penis, fulfilling this criterion.

**Figure 1:**
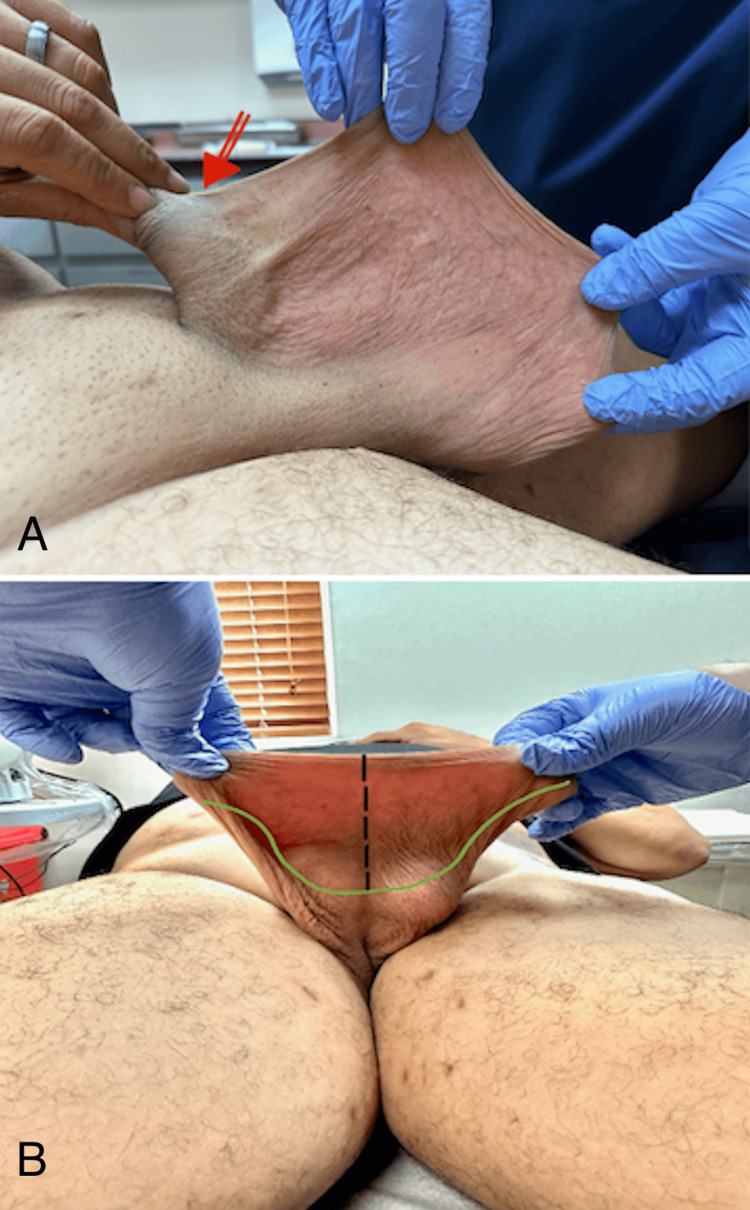
Preoperative assessment of penoscrotal webbing and planned surgical incision. (A) The red arrow highlights the patient’s severe penoscrotal webbing, which resulted from excessive prepuce removal during prior circumcision, leading to significant scrotal skin being pulled onto the penile shaft. (B) Preoperative view of the incision site, demonstrating the modified transverse check mark-shaped incision. The black dashed line represents the midline, while the green markings outline the planned excision area.

On examination, there was no recurrence of hydrocele, but the patient had marked scrotal laxity and prominent PSW (Figure 1). The excess posterior scrotal skin showed signs of irritation and cobblestoning, contributing to his discomfort. Due to his symptoms, the patient consented to undergo scrotal reduction surgery combined with ventral phalloplasty.

Surgical approach

In our patient, we designed a novel modified transverse check-mark-shaped incision to comprehensively address both scrotal laxity and penoscrotal webbing. Miranda-Sousa et al. (2007) originally described a check-mark-shaped incision, an asymmetric V-shaped design with a longer inferior (caudal) segment, to minimize redundant skin during penile prosthesis surgery and prevent the formation of dog ears at closure [7]. However, their technique primarily focused on removing the penoscrotal webbing, leaving scrotal laxity unaddressed. Because this approach alone did not fully correct the redundant posterior scrotal skin in our patient, we modified the incision to achieve a more comprehensive correction of both scrotal laxity and penoscrotal webbing.

**Figure 2:**
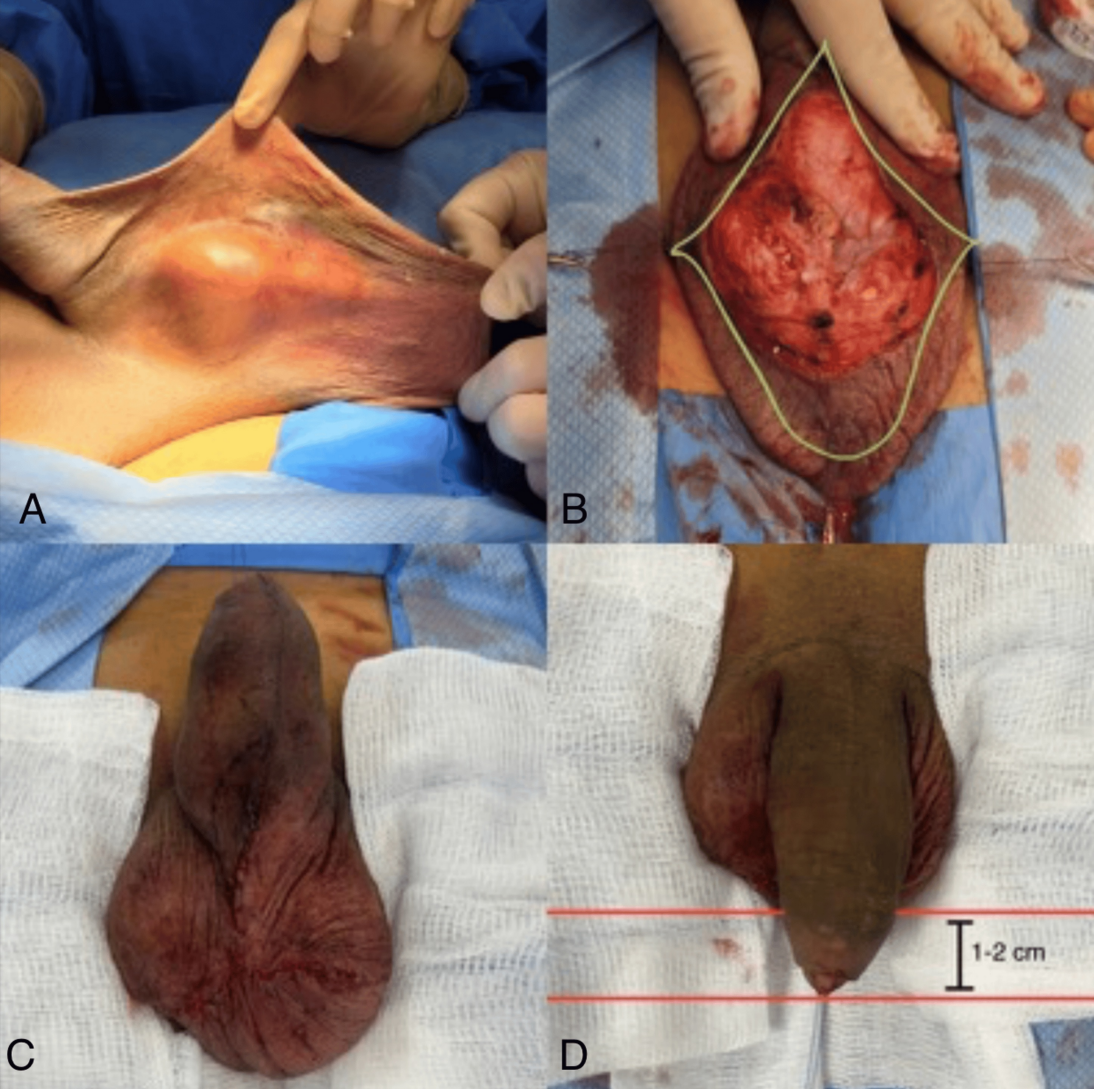
Preoperative, intraoperative, and postoperative stages of the scrotal reduction and ventral phalloplasty procedure. (A) The patient’s preoperative condition, showing severe scrotal laxity and penoscrotal webbing. (B) Intraoperative marking of the modified transverse check mark-shaped incision, outlining the excision area while preserving key anatomical structures. (C and D) The immediate postoperative outcome, demonstrating an improved scrotal contour and enhanced perception of penile length, with the glans positioned approximately 1-2 cm below the inferior scrotal border.

The surgical incision, as shown in Figure 2, involved a bilateral transverse check mark incision on the redundant scrotal tissue, extending to the ventral penile shaft and carried to the base of the penis at the newly proposed penoscrotal junction. The excess scrotal skin, including the previous hydrocelectomy incision, was excised without entering the tunica vaginalis bilaterally. The underlying dartos fascia was preserved to maintain vascularity and optimize wound healing.

Closure was performed using monofilament absorbable sutures, resulting in an inverted Y-shaped closure with a long segment along the median raphe. Care was taken to ensure the penile glans hung 1-2 cm below the inferior scrotal border. This approach effectively reduced excess posterior scrotal skin while improving both functional and cosmetic outcomes, addressing the patient’s primary concerns.

Postoperative course

The patient was discharged the same day with prescriptions for oral antibiotics, acetaminophen, and gabapentin. On postoperative day (POD) 1, a follow-up phone call confirmed that he had no complications and was managing mild discomfort with acetaminophen alone.

In-office evaluations on POD 5, 12, and 26 showed proper wound healing without signs of dehiscence, hematoma, or infection. The patient reported no need for narcotic pain management. By POD 7, he had resumed normal daily activities, and by POD 14, he resumed sexual activity.

At each follow-up visit, he expressed satisfaction with the improved scrotal contour, perception of increased penile length, and overall functional and cosmetic outcomes.

## Discussion

Excessive scrotal skin with increased laxity and PSW can significantly impact a patient’s quality of life, the perception of penile length, and both patient and partner sexual satisfaction. These conditions can arise following hydrocelectomy, orchiectomy, inguinal hernia repair, significant weight loss, and other procedures. Scrotal laxity is often associated with penoscrotal webbing, which may also result from excessive prepuce removal during circumcision, leading to scrotal skin being pulled onto the penile shaft. When evaluating an adult patient with symptomatic or bothersome scrotal laxity, it is essential to obtain a comprehensive medical history and perform a thorough scrotal and testicular examination to assess the severity of the condition and determine the most appropriate treatment approach [1].

Various surgical techniques have been described for scrotal reduction, including crescent-shaped horizontal resection, inverted-U incisions, and vertical ventral resection with horizontal closure. Ventral phalloplasty techniques include penoscrotal Z-plasties, Y-V penoscrotal junction closure, and transverse skin resection with ventral closure [1].

The modified transverse check mark incision offers several advantages over vertical scrotal resection. It allows the precise excision of excess scrotal tissue, resulting in improved scrotal contour and aesthetics [1,8,9]. Moreover, longitudinal closure using this incision stabilizes the proximal penile skin, preventing penile retraction and enhancing the perceived penile length, thereby improving patient satisfaction [7-9]. Studies have shown that 84% (36/43) of patients report an improved perception of penile length after this procedure [1,8,9].

Thomas and Navia (2021) advocate for vertical skin resection, arguing that it provides a more anatomical and aesthetically pleasing outcome by positioning the scar in the median raphe and better preserving scrotal sensitivity due to the lateral-to-medial course of the genital branch of the genitofemoral nerve and ilioinguinal nerves [1]. However, in cases with significant scrotal laxity and PSW, the modified novel check mark-shaped incision may be preferable due to its enhanced contouring effects and ability to optimize both functional and cosmetic outcomes [10].

## Conclusions

Scrotal reduction surgery with concomitant ventral phalloplasty is a safe and effective procedure that significantly enhances patient quality of life, genital aesthetics, and functional outcomes. This technique, using a modified transverse check mark-shaped incision, provides superior scrotal contouring, enhances perceived penile length, and optimizes wound healing by reducing closure tension. As the awareness of post-surgical scrotal laxity increases, further research and long-term follow-up are necessary to refine surgical techniques and establish standardized treatment guidelines.
